# Scaffolds Loaded with Dialdehyde Chitosan and Collagen—Their Physico-Chemical Properties and Biological Assessment

**DOI:** 10.3390/polym14091818

**Published:** 2022-04-29

**Authors:** Sylwia Grabska-Zielińska, Judith M. Pin, Beata Kaczmarek-Szczepańska, Ewa Olewnik-Kruszkowska, Alina Sionkowska, Fernando J. Monteiro, Kerstin Steinbrink, Konrad Kleszczyński

**Affiliations:** 1 Department of Physical Chemistry and Physicochemistry of Polymers, Faculty of Chemistry, Nicolaus Copernicus University, Gagarin 7, 87-100 Toruń, Poland; olewnik@umk.pl; 2 Department of Dermatology, University of Münster, Von-Esmarch-Str. 58, 48149 Münster, Germany; j_pin001@uni-muenster.de (J.M.P.); kerstin.steinbrink@ukmuenster.de (K.S.); konrad.kleszczynski@ukmuenster.de (K.K.); 3 Department of Biomaterials and Cosmetics Chemistry, Faculty of Chemistry, Nicolaus Copernicus University, Gagarin 7, 87-100 Toruń, Poland; beata.kaczmarek@umk.pl (B.K.-S.); alinas@umk.pl (A.S.); 4 i3S—Instituto de Investigação e Inovação em Saúde, Universidade do Porto, 4200-180 Porto, Portugal; fjmont@i3s.up.pt; 5 INEB—Instituto de Engenharia Biomédica, Universidade do Porto, 4200-180 Porto, Portugal; 6 FEUP—Faculdade de Engenharia, Universidade do Porto, 4200-465 Porto, Portugal

**Keywords:** chitosan dialdehyde, collagen, bioengineering, cutaneous cells, scaffolds

## Abstract

In this work, dialdehyde chitosan (DAC) and collagen (Coll) scaffolds have been prepared and their physico-chemical properties have been evaluated. Their structural properties were studied by Fourier Transform Infrared Spectroscopy with Attenuated Internal Reflection (FTIR–ATR) accompanied by evaluation of thermal stability, porosity, density, moisture content and microstructure by Scanning Electron Microscopy—SEM. Additionally, cutaneous assessment using human epidermal keratinocytes (NHEK), dermal fibroblasts (NHDF) and melanoma cells (A375 and G-361) was performed. Based on thermal studies, two regions in DTG curves could be distinguished in each type of scaffold, what can be assigned to the elimination of water and the polymeric structure degradation of the materials components. The type of scaffold had no major effect on the porosity of the materials, but the water content of the materials decreased with increasing dialdehyde chitosan content in subjected matrices. Briefly, a drop in proliferation was noticed for scaffolds containing 20DAC/80Coll compared to matrices with collagen alone. Furthermore, increased content of DAC (50DAC/50Coll) either significantly induced the proliferation rate or maintains its ratio compared to the control matrix. This delivery is a promising technique for additional explorations targeting therapies in regenerative dermatology. The using of dialdehyde chitosan as one of the main scaffolds components is the novelty in terms of bioengineering.

## 1. Introduction

Collagen (Coll) is one of the most important biopolymers, belonging to the group of proteins [1,2]. It is found in the skin, bone and cartilage tissue, tendons, endothelial vessels, and in the extracellular matrix (ECM) [3,4,5]. The collagen family consists of 29 distinct collagen types. They are divided into four classes based on existence of various α chains, isoforms of particles, supermolecular structures of each collagen type, differences in the expressions of genes involved in protein biosynthesis and post-translational modifications of collagens [2,3]. Thus, the class of collagen depends on its structural and composition properties [2].

Collagen is widely used in many areas including biomaterials, tissue engineering, drug delivery systems, cosmetology, pharmacy, and the food industry [4,6,7,8]. As a biomaterial, collagen exerts numerous advantages such as biodegradability and bioresorbability, non-toxicity and biocompatibility, non-antigenicity, as well as synergy with bioactive components. Moreover, the possibility of its formulation in a number of different forms, it is easily modifiable to produce materials as desired by utilizing its functional groups, compatibility with synthetic polymers [7]. Additionally, it also has some disadvantages, i.e., a high cost of pure type I collagen, variability of isolated collagen (e.g., crosslink density, fiber size, trace impurities, etc.), hydrophilicity which leads to swelling and a more rapid release of substances incorporated to material, and variability in enzymatic degradation rate as compared with hydrolytic degradation. Furthermore, complex handling properties, side effects such as bovine spongeform encephalopathy (BSF), mineralization, low stability under high temperature or presence of enzymes [4,7]; therefore, the structure of pure collagen requires stabilization and modification [4,7].

The most commonly used methods to modify collagen materials are mixing with natural (chitosan [9], hyaluronic acid [10], silk fibroin [11], elastin [12], keratin [13]) or synthetic polymers (poly(vinyl pyrrolidone) [14], poly(vinyl alcohol) [15], poly(ethylene glycol) [16], poly(ethylene oxide) [16]), and cross-linking with chemical (EDC/NHS [17], dialdehyde starch [18], glutaraldehyde [19], genipin [20]), physical (temperature [21], UV light [22]) or enzymatic (microbial transglutaminase [4,23]) factors.

**Figure 1:**
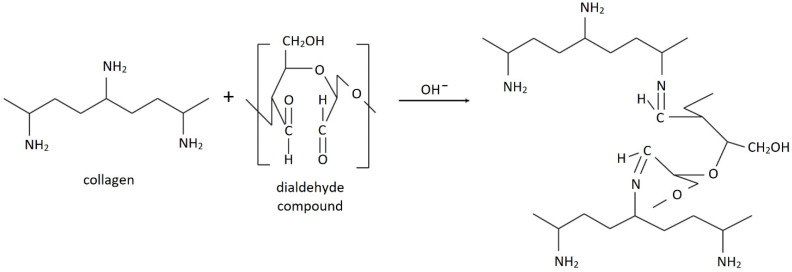
The mechanism of collagen and dialdehyde compound reaction (based on collagen and dialdehyde cellulose example from Pietrucha and Safandowska’s work [32]).

Dialdehyde chitosan is the compound obtained from chitosan by oxidation with sodium or potassium periodate. The process of periodate oxidation endows chitosan with multiple functional aldehyde groups. The dialdehydes, including dialdehyde chitosan, are considered as a safe additives and green cross-linking agents for various biomaterials and nanomaterials. They also can be used as a biological tissue fixation and tanning agents [24,25,26,27]. The use of dialdehyde compounds for materials’ modification is a good route since dialdehydes react readily with functional groups from other polymers. Dialdehyde starch [18,28,29], dialdehyde carboxymethyl cellulose [30], dialdehyde nanocellulose [31], dialdehyde cellulose [32], dialdehyde alginate [33,34] and dialdehyde chitosan [24,27] have been used to cross-link collagen materials. Thus, Figure 1 shows the mechanism of the reaction between collagen and a dialdehyde compound.

Pietrucha and Safandowska [32] have studied the physicochemical properties of silver carp collagen modified by dialdehyde cellulose, and they reported a marked increase in thermostability of collagen structure and the improvement of the mechanical strength of the modified materials. Hu et al. [33] described the interaction between collagen and alginate dialdehyde (ADA) used as naturally derived cross-linker, and they indicated that the ADA addition could improve the properties of collagen-based materials such as thermal stability and hydrophilicity. Additionally, the dialdehyde compound resulted in the aggregation of collagen molecules, not destroying the triple helix conformation of collagen and it has a positive effect on cells proliferation at a certain content of ADA [33]. Yu et al. [34] also used dialdehyde alginate to modify collagen and proved that ADA significantly improving swelling, rheological behaviors and capability to resist against type I collagenase. Concerning dialdehyde chitosan as a cross-linking agent, Wanli et al. [35] used it for collagen fibers cross-linking. They reported that the thermal denaturation temperature of collagen fiber rose with increasing oxidation degree of chitosan dialdehyde, and the porosity of collagen fiber was reduced accordingly [35]. Liu et al. [27] also worked with dialdehyde chitosan as cross-linking agent for collagen materials and concluded that introducing DAC into collagen may be favorable for cellular growth, adhesion and proliferation. According to their report, chitosan dialdehyde might be an ideal cross-linking agent for the chemical fixation of collagen [27]. On the other hand, Bam et al. [24] designed biostable scaffolds based on collagen cross-linked with dialdehyde chitosan in the presence of gallic acid (GA), and observed that the formed stable Schiff’s base between collagen and DAC with GA had significant effects in improving microstructural integrity. Additionally, the texture, thermal and structural properties, biostability, swelling and water uptake have been improved after the introduction of DAC in the scaffolds [24].

In this work, we decided to use dialdehyde chitosan as one of the scaffolds components. Usually, as we considered above, dialdehydes generally were used as additives to mixtures of biopolymers or to pure biopolymers, that is as cross-linking agents [24,25,26,27], not as one of the main components of scaffolds. The aim of this work was to obtain and characterize materials based on collagen with chitosan dialdehyde (DAC) in various compositions (DAC/Coll: 80/20, 50/50 and 20/80). The use of dialdehyde chitosan as a component of collagen materials, not a cross-linking agent is a novelty. There is only one report, where dialdehyde chitosan is mixed as component of scaffold. Mixtures with hyaluronic acid and the results of their physico-chemical characterization were described in our previous reports [36,37].

## 2. Materials and Methods

### 2.1. Materials

Collagen (Coll) and dialdehyde chitosan (DAC) were obtained in-house. Collagen was prepared from tail tendons of young rats following our previously reported method [38]. Dialdehyde chitosan was obtained by one-step synthesis following the method described by Bam et al. [24] with slight modifications. The synthesis of dialdehyde chitosan was previously described by this research group [36]. Reagents purchased form Sigma-Aldrich (St. Louis, MO, USA): chitosan (DD = 78%), acetic acid, HCl, acetone, isopropanol, sodium periodate, Minimum Essential Medium Eagle (MEM) (1000 mg/L), 1% penicillin-streptomycin solution, 3-(4,5-dimethylthiazol-2-yl)-2,5-diphenyltetrazolium bromide (MTT), l-glutamine (200 mM), and 0.05% trypsin/0.53 mM EDTA solution. Fetal bovine serum was purchased from Thermo Fisher Scientific (Waltham, MA, USA). Human epidermal keratinocytes (NHEKs) and human dermal fibroblasts (NHDFs) were supplied by PromoCell (Heidelberg, Germany) and American Type Culture Collection (ATCC) (Manassas, VA, USA), respectively. Human melanoma cell i.e., amelanotic A375 and G-361 cell lines supplied by ATCC (Manassas, VA, USA).

### 2.2. Obtaining the Scaffolds

Chitosan dialdehyde was dissolved in water and collagen was dissolved in 0.1 M acetic acid at 1% concentration separately. They were mixed in different weight ratios: 80/20, 50/50 and 20/80. The scaffolds based on pure collagen were treated as control samples. Solutions were mixed with a magnetic stirrer for 1 h and the obtained mixtures were poured into 24-well polystyrene culture plates, frozen, and lyophilized (−20 °C, 100 Pa, 48 h, ALPHA 1–2 LDplus, CHRIST, Ostreode am Harz, Germany).

### 2.3. Structural Studies—Attenuated Total Reflectance–Fourier Transform Infrared Spectroscopy (FTIR-ATR)

Nicolet iS10 spectrometer equipped with an attenuated total reflectance (FTIR–ATR) device with a germanium crystal (Nicolet iS10, Thermo Fisher Scientific, Waltham, MA, USA) was used to analyze the chemical structure of the obtained scaffolds. The spectra were evaluated in the range of 600–4000 cm^−1^. All spectra were recorded with the resolution of 4 cm^−1^ with 64 scans.

### 2.4. Thermal Stability

Thermogravimetric analyses were performed at a heating rate of 10 °C/min (20–600 °C) under nitrogen atmosphere, by using TA Instruments SDT 2960 Simultaneous TG-DTG (TA Instruments Manufacturer, Eschborn, Germany). From thermogravimetric curves, the characteristic temperature at a maximum decomposition rate of the investigated composites was determined.

### 2.5. Determination of Density, Porosity and Water Content

The liquid displacement method with isopropanol was used to measure density and porosity of the scaffolds. A fragment of the sample with a known weight was immersed in a cylinder with a known volume of isopropanol for 3 min. The density was calculated using the following Equation (1):

(1) $$d\;\left[\frac{\mathrm{mg}}{{\mathrm{cm}}^{3}}\right]=\frac{W}{V_{2}-V_{3}}\cdot 100\%$$

where W—weight of sample (mg), V_2_—total volume of isopropanol with the isopropanol impregnated sample (cm^3^), and V_3_—volume of isopropanol after scaffold removal (cm^3^). The porosity was calculated using following Equation (2):

(2) $$\varepsilon \;\left[\%\right]=\frac{V_{1}-V_{3}}{V_{2}-V_{3}}\times 100\%$$

where V_1_—initial volume of isopropanol (cm^3^), and V_2_, V_3_—as above.

Gravimetric analysis was used to determine the water content of the samples. The water content of scaffolds was measured by drying samples at 105 °C until they reached a constant weight. The results were expressed as grams of water per 100 g of dry sample.

### 2.6. Scanning Electron Microscopy Imaging

The morphology of the samples was studied using a Scanning Electron Microscope (LEO Electron Microscopy Ltd., Cambridge, UK). Scaffolds were frozen in liquid nitrogen for a few minutes, cut with a razor blade and gold coated prior to the observation.

### 2.7. Cell Culture and Proliferation Ratio Assessment

Human epidermal keratinocytes (NHEKs) and human dermal fibroblasts (NHDFs) were supplied by PromoCell (Heidelberg, Germany); amelanotic A375 and G-361 cell lines were supplied by the American Type Culture Collection (ATCC) (Manassas, VA, USA), respectively. NHEKs were grown in Keratinocyte Growth Medium 2 supplemented with 1% penicillin-streptomycin solution while NHDFs were maintained in MEM medium supplemented with 10% (*v*/*v*) heat-inactivated fetal bovine serum, 2 mM of L-glutamine, and 1% (*v*/*v*) streptomycin-penicillin solution. Melanoma cells were maintained in MEM medium supplemented with 10% (*v*/*v*) heat-inactivated fetal bovine serum, 2 mM of L-glutamine, and 1% (*v*/*v*) streptomycin-penicillin solution. Cells were seeded in 24-wells plates at the density of 0.5 × 10^5^ cells/well and were allowed to attach to the surface of the scaffolds for 24 h. Afterwards, cells were cultured in supplemented culture medium in a humidified 5% CO_2_ atmosphere at 37 °C for 96 h while the culture medium was exchanged every 48 h. Differences in cell viability were assessed using the MTT assay. MTT (5 mg/mL in 1 × PBS) was prepared in the respective culture medium (the final dilution, 1:10), 100 μL of assay reagent was added to each well, and cells were subsequently incubated for 3 h in a humidified atmosphere of 5% CO_2_ at 37 °C. The resultant formazan crystals were dissolved using 100 μL isopropanol/0.04 N HCl, absorbance was measured at λ = 595 nm using the BioTek ELx808™ microplate reader (BioTek Instruments, Inc., Winooski, VT, USA), and the results were normalized to the control cells.

### 2.8. Statistics

Statistical analysis of the data was completed using commercial software (GraphPad Prism 8.0.1.244, GraphPad Software, San Diego, CA, USA). The results were presented as a mean ± standard deviation (S.D.) and were statistically analyzed using one-way analysis of variance (one-way ANOVA). Multiple comparisons between the means were performed with the statistical significance set at *p* ≤ 0.05. Results from mechanical tests, density, porosity and water content measurements were subjected to statistical analysis.

For cell culture and proliferation ratio assessment, data were expressed as pooled means + S.D. of six independent experiments (*n* = 6). Statistically significant differences between results were determined by the univariate analysis of variance (ANOVA) or the Student’s *t*-test and appropriate post hoc analysis (Tukey or Dunnett tests, accordingly). All the analysis are presented as percentage of the control sample and a *p* < 0.05 was considered as statistically significant.

## 3. Results and Discussion

### 3.1. Structural Studies—FTIR-ATR

**Figure 2:**
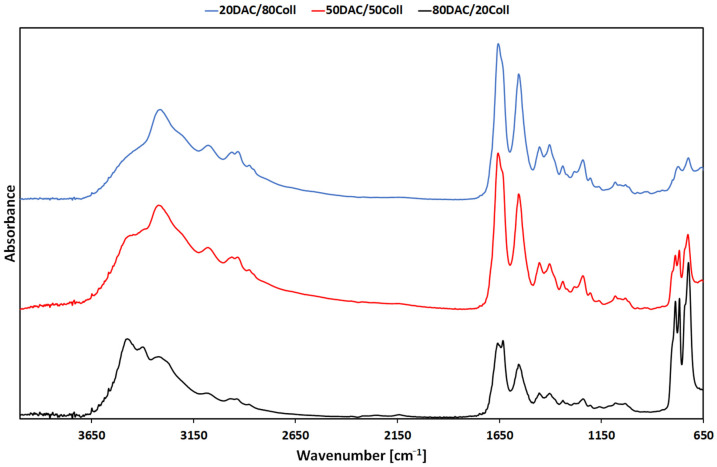
The FTIR-ATR spectra of DAC/Coll based materials.

To evaluate the molecular structure of DAC/Coll scaffolds and confirm possible interactions between dialdehyde chitosan and collagen, FTIR-ATR analysis was performed. Additionally, FTIR analysis allows to observe the presence of functional groups, that may be used to identify compounds and interactions between material components. The spectra of the obtained materials are shown in Figure 2. The structure of collagen [37,38,39,40,41] and dialdehyde chitosan [25,26,27,42,43] have often been analyzed and described in the literature (Figure S1 in the Supplementary Materials). Herein, it was decided to record only the spectra of mixtures based on dialdehyde chitosan and collagen, as presented via the positions of their characteristic bands (Table 1). It may be observed that the position of Amide II and Amide III does not depend on the DAC/Coll weight ratio. However, the Amide A and Amide B band positions are shifted. This suggests that they participate in the formation of hydrogen bonds between these materials. Additionally, the C–OH peak was not observed in the spectra with the lowest DAC content. The –C=N peak was noticed only for the material composition with the highest DAC content. In the other two materials (20DAC/80Coll and 50DAC/50Coll), this peak of stretching vibration (around 1630–1640 cm^−1^) formed by Schiff base reaction overlapped with the C=O stretching in Amide I [44]. Based on these observations, our results indicated that dialdehyde chitosan was successfully introduced into the collagen matrix.

### 3.2. Thermal Stability

The biopolymers are characterized by low denaturation temperature, and their thermal properties should be considered (Table 2). TG-DTG curves of collagen, 20DAC/80Coll and 80DAC/20Coll scaffolds are shown in Figure S2 and S3 in the Supplementary Materials.

Two regions in DTG curves could be distinguished in each type of scaffolds. The first one may be correlated with the elimination of water molecules present in the scaffolds [36]. For scaffolds made of pristine collagen, it was 47.5 °C. For materials based on dialdehyde chitosan and collagen mixtures, the region responsible for water elimination, were observed at much higher temperature, namely 147.0 °C for 20DAC/80Coll scaffolds and approximately 147 °C for 50/50 and 80/20 DAC/Coll materials. Thus, no significant differences between scaffolds with different DAC/Coll compositions were observed, but a significant improvement in thermal stability was noticed for DAC/Coll based materials over pristine collagen-based matrices. The second region in DTG curves may be assigned to the degradation of the polymeric structure of the materials components [36] and the fast volatilization of the polymer segment due to the thermal scission of the polymer backbone [44]. T_max_ (2) for collagen materials was comparable with T_max_ (2) for DAC/Coll samples, and it was within the limits of 307.3 to 326.7 °C. Similar results were obtained by Liu et al. [27] who showed that the addition of oxidized chitosan resulted in the increase in maximum temperature of the first and second stages. Additionally, the addition of chitosan dialdehyde to cellulose increased the thermal stability of the obtained films [44]. In summary, for materials made of dialdehyde chitosan and collagen-based mixtures, the temperature below 146 °C is safe and does not cause material degradation. The Schiff base reactions (what was reported in Section 1—Figure 1 and Section 3.1. Structural studies—FTIR–ATR) that occurred between dialdehyde chitosan and collagen significantly improved the thermal stability of the scaffolds (Figure 1) [44].

### 3.3. Density, Porosity and Water Content

**Figure 3:**
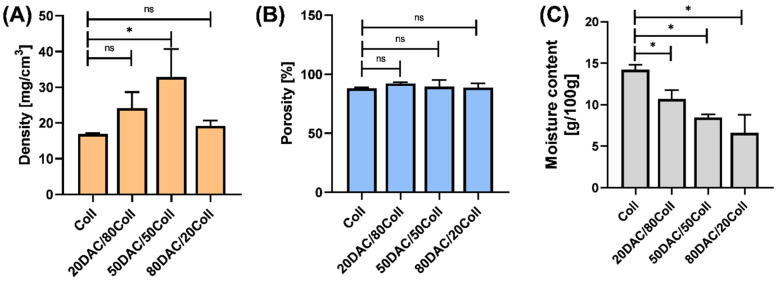
Results for (**A**) density, (**B**) porosity and (**C**) moisture content of DAC/Coll scaffolds. Statistically significant differences versus pristine collagen scaffolds were indicated as follows: * *p* < 0.05; ns—not significant.

Parameters such as density, porosity or moisture content are important from the point of view of using the material in tissue engineering. They were determined and the results are shown in Figure 3.

Namely, the porosity (Figure 3B) of investigated materials ranged from 88.08 ± 0.64% (Coll) to 92.27 ± 0.99% (20DAC/80Coll). No statistically significant differences between control (Coll) and other materials were observed. Potential material targeting tissue engineering should be characterized by porosity of about 90% [45,46,47]. Here, porosity of our assessed matrices was higher or almost 90% and this means that they met the above requirement. The content of dialdehyde chitosan in the scaffolds had no major influence on the porosity of the materials but the addition of dialdehyde chitosan to collagen may affect the density of the material, which is in agreement with Pietrucha and Safandowska [32].

Furthermore, the highest density was observed for 50DAC/50Coll scaffolds. No statistically significant differences between pristine collagen scaffolds and 20DAC/80Coll and 80DAC/20Coll have been noticed (Figure 3A). With increasing dialdehyde chitosan to collagen, the material density increased until at the weight ratio 50DAC/50Coll.

Concerning moisture content (Figure 3C), it may be seen that it decreased with increasing dialdehyde chitosan to collagen ratio, and it reached 14.22 ± 0.34 g/100 g for collagen and 6.62 ± 1.27 g/100 g for 80DAC/20Coll scaffold. The moisture content of pure collagen materials was the same as in our previous study, where collagen, collagen and silk fibroin as well as collagen, silk fibroin and chitosan scaffolds were cross-linked with dialdehyde starch [47]. In the above-mentioned study, it was concluded that in the case of materials modified with a dialdehyde compound, the moisture content decreased in comparison with the moisture content found in the unmodified material [47]. It suggests that collagen has more polar character than chitosan as it has more hydrophilic groups for a given chain length. Only with pristine collagen the moisture content for the modified and unmodified material was 14.17 ± 1.36 g/100 g and 13.10 ± 0.79 g/100 g, respectively. Nevertheless, such a small difference could be due to the fact that in that study only a 10% addition of dialdehyde compound to the polymer was used [47].

### 3.4. Morphological Studies—SEM

**Figure 4:**
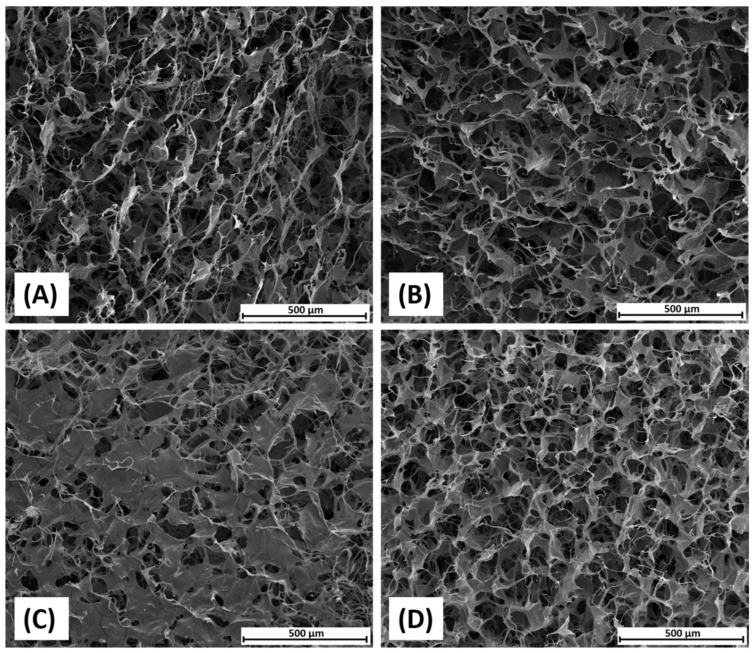
The SEM pictures of collagen (**A**); 20DAC/80Coll (**B**); 50DAC/50Coll (**C**); 80DAC/20Coll (**D**) scaffolds. Magnification 200×.

SEM imaging was used to assess the morphological structure of scaffolds. The SEM images show the inner region of the scaffold after cutting it with a scalpel, as shown in Figure 4. Namely, the heteroporous structure of collagen and dialdehyde chitosan/collagen materials were well-ordered. After freeze-drying process where solvents (water and 0.1 M acetic acid) act as pore-forming agents (porogens), the materials were characterized by pores with variable size and geometry. The scaffold morphology was related to the feed ratio of dialdehyde chitosan to collagen. As for 80DAC/20Coll and 20DAC/80Coll materials, the sponges had structures very similar to that of native collagen. 80DAC/20Coll materials presented the best microstructure. Pores were most regular and most similar to each other in shape and size that diameter was approximately 140 µm for collagen, 133 µm for 20DAC/80Coll, 90 µm for 50DAC/50Coll, and 75 µm for 80DAC/20Coll.

About 50DAC/50Coll, the material was characterized by a heterogenous structure, and it could be a result of the excessively fast cross-linking between collagen and dialdehyde chitosan. In this case, the pores were irregular, more closed and unequal. A similar observation was reported by Ding et al. [48] where collagen materials were cross-linked by dialdehyde cellulose. This is consistent with the results of density (Figure 3), e.g., the highest density was visible, and it was 32.89 ± 4.50 mg/cm^3^.

Bam et al. [24] reported a little bit more flattened structure of DAC modified collagen than pure collagen scaffold. Mu et al. [18] studied several physico-chemical properties of collagen materials modified with dialdehyde starch (DAS) with weight ratios of DAS/Coll: 1:100, 1:70 and 1:10, and they observed that fibrous surface structures were more pronounced at low DAS content, while integrally lamellar aggregates appeared as DAS increased. One may say that this coincides with our results if we look at the materials 50DAC/50Coll (C) and 80DAC/20Coll (D). They also reported [18] that the thickness of the pore wall increased with the content of DAS, and their cryogels became more solid with higher DAS content.

### 3.5. Cellular Assessments Using Cutaneous Models

**Figure 5:**
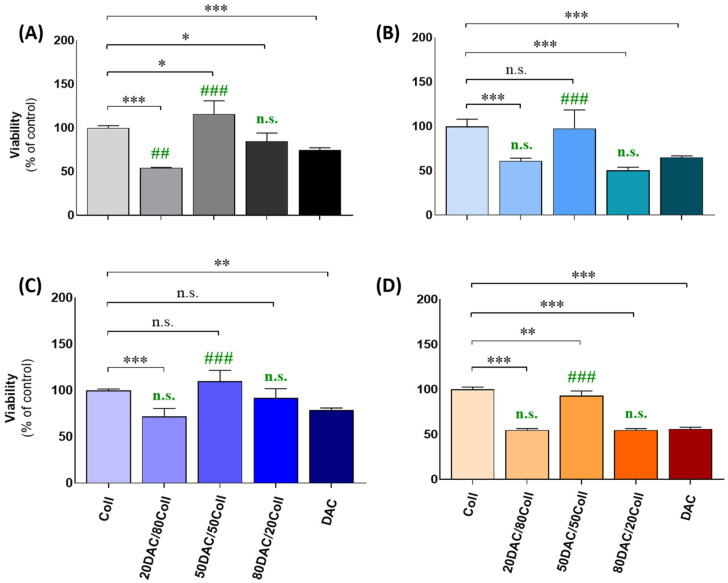
Assessment of proliferation rate in human cutaneous cell lines. Human cells from epidermal keratinocytes (NHEKs, **A**), dermal fibroblasts (NHDFs, **B**) as well as human amelanotic melanoma models, i.e., A375 (**C**) and G-361 (**D**) were seeded on subjected scaffolds loaded with respective components, cultured for 96 h, and viability was assessed using the MTT viability assay as described in Section 2. Data are presented as mean S.E.M. (*n* = 6), expressed as a percentage of the control cells cultured on matrices with collagen. Statistically significant differences versus collagen-contained scaffolds were indicated as follows: * *p* < 0.05, ** *p* < 0.01, *** *p* < 0.001 while changes versus scaffolds with DAC alone were indicated as ## *p* < 0.01 and ### *p* < 0.01 and selected with green color; n.s.—not significant.

We performed the proliferation rate assessment (Figure 5) using selected cutaneous cell models, i.e., human epidermal keratinocytes and dermal fibroblasts providing the new insight into a considerable improvement of wound dressing, re-epithelization or therapeutic approaches for skin. Comparatively, we also tested melanoma cell lines used as reference cellular models to confirm the responses of primary cell lines as it was presented in our latest studies [36,49,50]. Within all investigated cell lines, we observed similar pattern of regulation where 20DAC/80Coll scaffolds caused prominent drop in cell proliferation, ranging from 29% to 46% versus scaffolds containing only collagen. Furthermore, increased content of DAC (50DAC/50Coll) either significantly induced the proliferation rate or maintained its ratio compared to the control matrix. A similar response was noticed when comparing scaffolds loaded only with DAC and those with respective addition of collagen. Namely, 50DAC/50Coll scaffolds enhanced proliferation rate versus DAC alone by 40% (NHEKs, Figure 5A), 33% (NHDFs, Figure 5B), 30% (A375, Figure 5C), 37% (G361, Figure 5D) while cells cultured on 80DAC/20Coll matrices revealed slight but not statistically significant increase versus DAC. The composition of 50DAC/50Coll provides the most suitable environment for cells as for them the cell viability is the highest for each type of cell line. It may be assumed that both collagen and dialdehyde chitosan are valuable as matrices.

Obtained results are in line with other physico-chemical properties enclosed in this study. Nevertheless, further assessments are utmost needed to understand the correlation between cell proliferation and the composition of scaffolds targeted in wound healing.

## 4. Conclusions

In conclusion, we have successfully prepared dialdehyde chitosan/collagen scaffolds. The dialdehyde chitosan have been prepared by a fast, not complicated, and cheap one step process. The materials have been obtained by a freeze-drying method.

The obtained scaffolds have been characterized by two regions in DTG curves, which were responsible for water molecules elimination and polymeric structure degradation in polymeric materials. The scaffold had a porous structure with a porosity around 90%, which is adequate for bioengineering applications. The highest material density was observed for 50DAC/50Coll scaffold, which is in agreement with SEM micrographs, where irregular and more closed structure of scaffold have been shown. Human epidermal keratinocytes (NHEK), dermal fibroblasts (NHDF) and reference melanoma cells (A375 and G-361) have been used for biological assessment of the obtained materials and that test led to the conclusion that increased content of dialdehyde chitosan either maintained or significantly increased the proliferation rates when compared to collagen scaffold. Thereby, it may be assumed that the most suitable material is in the composition 50DAC/50Coll.

Further tests to characterize dialdehyde compounds/collagen materials could be performed as a next step of this area of research. However, it is believed that the addition of chitosan dialdehyde to collagen may find potential use in the preparation of biopolymeric scaffolds for biological applications.

## Figures and Tables

**Table 1:** The positions of characteristic bands of DAC/Coll based scaffolds.

Specimen	Amide A	Amide B	CH_3_	C–OH	Amide I	C=N	Amide II	Amide III
20DAC/80Coll	3313	3077	2932	−	1656	−	1556	1241
50DAC/50Coll	3306	3078	2934	1730	1656	−	1556	1241
80DAC/20Coll	3322	3086	2938	1731	1658	1632	1556	1240

**Table 2:** Results of DTG analysis with temperatures of maximum peaks.

Specimen	T_max_ (1) (°C)	T_max_ (2) (°C)
100Coll	47.5	324.3
20DAC/80Coll	147.0	326.7
50DAC/50Coll	147.1	314.3
80DAC/20Coll	147.6	307.3

## Data Availability

The data presented in this study are available on request from the corresponding author. The data are not publicly available due to projects realization.

## Supplementary Materials

The following supporting information can be downloaded at: https://www.mdpi.com/article/10.3390/polym14091818/s1, Figure S1. The FTIR spectra of (A) collagen and (B) dialdehyde chitosan, Figure S2. The TGA-DTA curves of collagen, Figure S3. The STG curves of 20DAC/80Coll and 80DAC/20Coll scaffolds.
