# Supplementary material for: Scaffolds Loaded with Dialdehyde Chitosan and Collagen—Their Physico-Chemical Properties and Biological Assessment
Source: Polymers (Basel). 2022 Apr 29;14(9):1818. doi: 10.3390/polym14091818 (PMC9103159; doi:10.3390/polym14091818)
Supplement: Supplementary file 1 [file polymers-14-01818-s001.zip › polymers-1682796-supplementary.pdf]

polymers

MDPI

*Supplementary Materials*

# Scaffolds Loaded with Dialdehyde Chitosan and Collagen—Their Physico-Chemical Properties and Biological Assessment

**Sylwia Grabska-Zielińska [1,*,†], Judith M. Pin [2,†], Beata Kaczmarek-Szczepańska [3], Ewa Olewnik-Kruszkowska [1], Alina Sionkowska [3], Fernando J. Monteiro [4,5,6], Kerstin Steinbrink [2], Konrad Kleszczyński [2]**

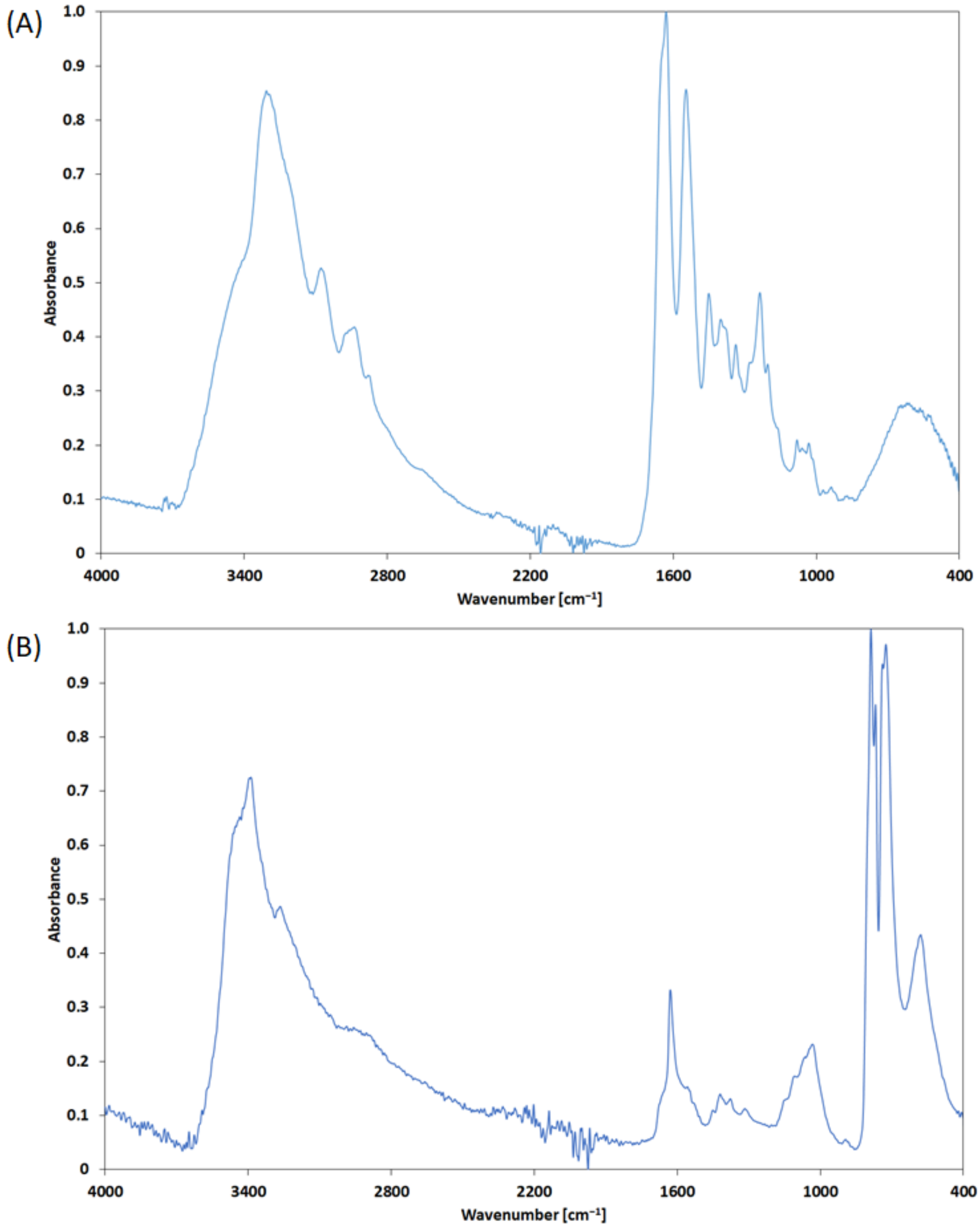


**Figure S1.** The FTIR spectra of (A) collagen and (B) dialdehyde chitosan.

*Polymers* **2022**, *14*, 1818. https://doi.org/10.3390/polym14091818 www.mdpi.com/journal/polymers

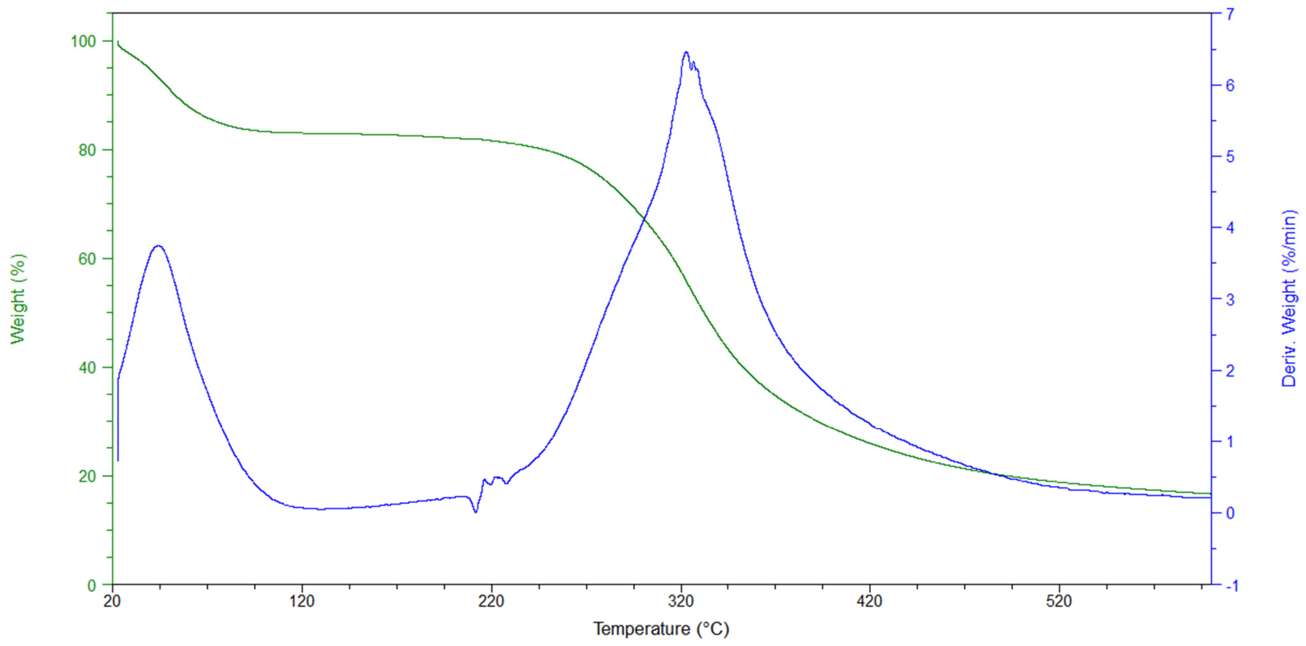


**Figure S2.** The TGA-DTA curves of collagen.

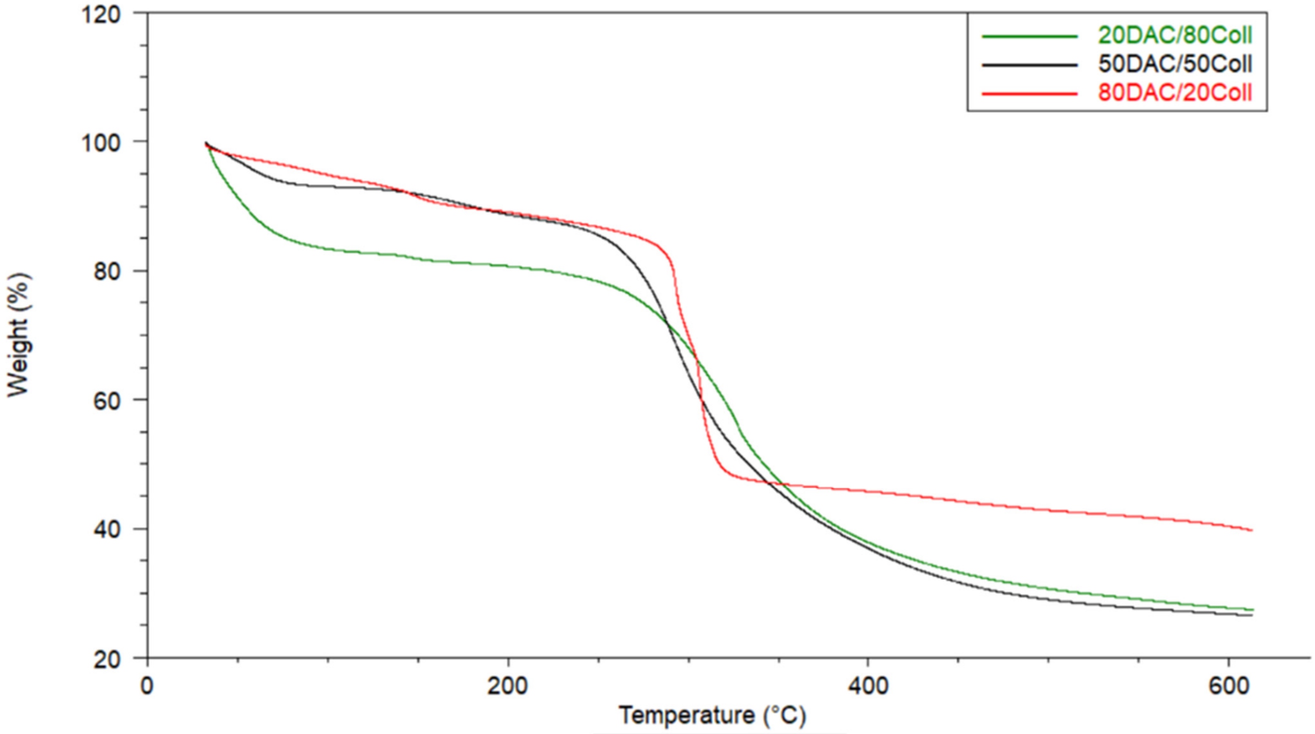


**Figure S3.** The DTG curves of 20DAC/80Coll and 80DAC/20Coll scaffolds.
